# Acute but Not Permanent Effects of Propranolol on Fear Memory Expression in Humans

**DOI:** 10.3389/fnhum.2019.00051

**Published:** 2019-02-21

**Authors:** Anastasia Chalkia, Jeroen Weermeijer, Lukas Van Oudenhove, Tom Beckers

**Affiliations:** ^1^Centre for the Psychology of Learning and Experimental Psychopathology, Department of Psychology, KU Leuven, Leuven, Belgium; ^2^Leuven Brain Institute, KU Leuven, Leuven, Belgium; ^3^Center for Contextual Psychiatry, Department of Neurosciences, KU Leuven, Leuven, Belgium; ^4^Laboratory for Brain-Gut Axis Studies, Translational Research Centre for Gastrointestinal Disorders, Department of Chronic Diseases, Metabolism, and Ageing, KU Leuven, Leuven, Belgium

**Keywords:** fear conditioning, extinction, reconsolidation, post-reactivation amnesia, state dependency, propranolol

## Abstract

Experimental evidence in humans and non-human animals suggests that the administration of propranolol shortly after the retrieval of an emotional memory can lead to an attenuation of its later expression, a phenomenon known as post-reactivation amnesia. Using more potent amnestic drugs, post-reactivation amnesia has been shown in animals to be reversible by re-administration of the drug prior to memory retention testing. The latter finding suggests that, at least under some circumstances, post-reactivation amnesia may not reflect a disruption of reconsolidation (i.e., a memory storage deficit) but an acquired state-dependency of memory expression (i.e., a memory retrieval deficit that is relieved when the drug state is recreated during testing). We conducted a double-blind, placebo-controlled study to investigate whether the previously established amnestic effects of post-reactivation propranolol administration on memory retention in humans may similarly reflect a retrieval deficit. In four groups of participants, fear memories were first established through differential fear conditioning. One day later, a single presentation of the CS+ without shock was used to reactivate the memory in three of the four groups, followed by the administration of 40 mg Propranolol HCl (Groups PrPl and PrPr) or placebo (Group PlPl). Memory was not reactivated in the fourth group (Group NR). Another 24 h later, Propranolol HCl (Group PrPr) or placebo (Groups PrPl, PlPl, and NR) was again administered, followed by a test of memory retention (extinction testing) and recovery (reinstatement testing). We did not observe any effects of post-reactivation propranolol on memory retention; conditioned responding was similar for all groups at the start of retention testing and similarly sensitive to recovery through reinstatement. We did observe an acute effect of propranolol administration on fear-potentiated startle responding during retention testing in Group PrPr, where participants exhibited attenuated startle responses during extinction testing but similar sensitivity to reinstatement as participants in the other groups. While our findings fail to corroborate previous reports of propranolol-induced post-reactivation amnesia in humans, they do point to acute effects of propranolol administration on extinction performance.

## Introduction

Accumulating experimental evidence in animals and humans points to the dynamic, rather than stable, nature of memory. In particular, it has been firmly established now that memory retrieval can destabilize a previously consolidated memory, inducing a labile state during which memory is sensitive to interference (for recent reviews see Beckers and Kindt, 2017; Lee et al., 2017; Elsey et al., 2018). Protein synthesis has been suggested to be one of the necessary processes for memory restabilization, hence, inhibition of protein synthesis during this period of destabilization has been widely investigated as a means to interfere with *reconsolidation* (Nader et al., 2000; Dunbar and Taylor, 2016). Blocking protein synthesis while a memory is malleable will theoretically prevent it from being restored, yielding retroactive amnesia in further tests of memory expression (i.e., memory storage deficit) (Tronson and Taylor, 2007). In animals, reports of reconsolidation blockade have involved administration of very powerful protein-synthesis inhibitors, such as anisomycin (Nader et al., 2000) or cycloheximide (Duvarci et al., 2005; Gisquet-Verrier et al., 2015). At doses tailored to yield observations of retrograde amnesia, these drugs would be severely toxic to humans (Gisquet-Verrier et al., 2015; Beckers and Kindt, 2017). Dȩbiec and LeDoux (2004), however, induced amnesia in rats by post-reactivation administration of propranolol, a β-adrenergic receptor antagonist. β-adrenergic signaling is known to play a crucial role in protein synthesis via the cAMP-PKA-CREB pathway (Tronson et al., 2012; Otis et al., 2015). Of importance, at a similar relative dose as used in rats, propranolol is perfectly safe for human use.

The findings of Dȩbiec and LeDoux (2004) inspired the first successful demonstration of pharmacological reconsolidation blockade in humans. In a study by Kindt et al. (2009), participants were differentially fear-conditioned to two spider images of which one (CS+) was reinforced with a mild shock unconditioned stimulus (US) to the wrist, whereas the other (CS-) was never followed by shock. Twenty-four hours later, they received either 40 mg Propranolol HCl or a placebo before being presented with one CS+ trial, without shock, in order to reactivate the conditioned fear memory established the day before. When their memory was tested on the third day, participants that had received propranolol prior to memory reactivation displayed a complete lack of differential fear-potentiated startle (FPS) responding and were insensitive to return of fear manipulations (i.e., reinstatement testing). Numerous follow-up studies of post-reactivation propranolol administration convincingly confirmed attenuated emotional responding in humans, while also demonstrating that it preserved declarative memory, i.e., knowledge of CS-US contingencies remained unaffected (Soeter and Kindt, 2010, 2011, 2015a,b; Sevenster et al., 2012, 2013, 2014; Kindt and Soeter, 2018; but see Bos et al., 2014; Schroyens et al., 2017).

As indicated above, the phenomenon of drug-induced *post-reactivation amnesia* has most often been interpreted to reflect a disruption of reconsolidation, and hence, a memory storage deficit (Duvarci and Nader, 2004; Hardt et al., 2009; Lee, 2009). Yet, recent evidence in animals has indicated that drug-induced amnesia may be reversible under some circumstances, in which case it could not reflect genuine memory erasure, but more likely points to a deficit in memory retrieval (Gisquet-Verrier et al., 2015). In a series of experiments, Gisquet-Verrier et al. (2015) conditioned rats in a one-trial inhibitory-avoidance protocol, in which escape from a white compartment was paired with shock. A brief exposure to the white compartment 48 h later was used to reactivate the threat memory. Cycloheximide, a potent protein-synthesis inhibitor, was systemically administered shortly following memory reactivation, and importantly, re-administered 48 h later prior to memory retention testing (group Cyclo-Cyclo). Control groups received either saline prior to testing (Cyclo-Sal), double administration of saline (Sal-Sal), or no memory reactivation (NR). Post-reactivation amnesia (i.e., absence of avoidance) was observed at test for the Cyclo-Sal group, in line with previous reports. Remarkably, however, re-administration of cycloheximide prior to testing completely reversed the amnesia and restored passive avoidance in the Cyclo-Cyclo group. Follow-up experiments investigating different routes of cycloheximide administration (i.e., intracerebroventricular, hippocampal) further supported these findings.

Challenging more than half a century’s worth of evidence not just on reconsolidation, but also on consolidation, Gisquet-Verrier et al. (2015) then went on to demonstrate that post-reactivation amnesia can be induced (and reversed) by a drug that does not affect protein synthesis. Rats were conditioned as in the aforementioned studies, and exposed to a sucrose solution prior to memory reactivation. After memory reactivation, they were injected with saline or Lithium Chloride (LiCl), an illness-producing drug that promotes conditioned taste aversion (CTA) learning but has no effect on protein synthesis. Rats that were administered LiCl after reactivation exhibited a clear memory deficit (lack of avoidance), as well as a CTA (aversion to sucrose). In line with the cycloheximide findings, re-administration of LiCl 48 h later, prior to retention testing, reversed the apparent amnesia. Of note, LiCl administration induced reversible retrograde amnesia without inhibiting protein synthesis, as evidenced by the fact that it supported the formation of a new CTA memory.

Whether storage or retrieval deficits are responsible for the observation of retrograde amnesia has been a long-standing controversy (for an extensive discussion see Riccio et al., 2006). Gisquet-Verrier et al. (2015) were not the first to advocate a retrieval view; this idea was first suggested by Miller and Springer (1972), who demonstrated that memory of a footshock can recover following electroconvulsive shock (ECS)-induced amnesia, by presenting a reminder footshock. Soon thereafter, Hinderliter et al. (1975) were able to reverse hypothermia-induced amnesia by recooling the animals before testing, and the idea of state-dependent retrieval emerged. According to this view, any intervention applied after memory retrieval, be it protein synthesis inhibition, ECS, or even hypothermia, can induce a change in internal state that becomes *integrated* in the active memory representation (Gisquet-Verrier and Riccio, 2012). This integration renders later memory expression state-dependent, and hence, if the internal state presented during or soon after retrieval is absent during subsequent testing, amnesia will be observed. However, if a reminder of the amnestic intervention that recreates the physiological state it induced is presented prior to testing, the observed amnesia should be reversed. In essence, then, the amnestic treatment acts as a salient cue necessary for later memory expression.

The integration hypothesis postulates that new information presented when memory is malleable becomes part of the memory, but also suggests that salience is a key factor (Gisquet-Verrier et al., 2015). Whereas it may seem plausible that administration of cycloheximide and similarly potent amnestic drugs can yield a discernable internal drug state that renders subsequent memory expression state-dependent, it is less clear that the integration hypothesis accounts for observations of post-reactivation amnesia following propranolol administration in humans. Propranolol, at the typical 40-mg dose administered in human studies (Kindt et al., 2009; Soeter and Kindt, 2010, 2011, 2015a; Sevenster et al., 2012), does not evoke a clearly discernable change in internal state, with participants in those studies typically failing to detect whether they had received propranolol or placebo (see, e.g., Kindt et al., 2009). While this need not rule out the possibility of state-dependent retrieval, given the evidence that physiological cues below the threshold of awareness can modulate emotional processing (e.g., Van Oudenhove et al., 2011; Azevedo et al., 2017), it arguably does render an account of post-reactivation amnesia in terms of a state-dependent retrieval deficit less obvious.

**FIGURE 1:**
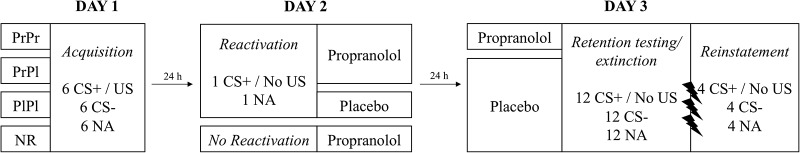
Overview of the experimental design.

We conducted a double-blind, placebo-controlled study to investigate whether previously reported amnestic effects of post-reactivation propranolol administration on fear memory retention in humans could reflect a retrieval deficit, as proposed by the integration hypothesis (see Figure 1 for an overview of the experimental design). First, we established fear memories through differential fear conditioning. Twenty-four hours later, a single presentation of the CS+ without shock was used to reactivate the memory. Memory reactivation was followed by the administration of 40 mg Propranolol HCl (Groups PrPl and PrPr) or placebo (Group PlPl). Memory was not reactivated in a fourth group (Group NR). Propranolol HCl (Group PrPr) or placebo (Groups PrPl, PlPl, and NR) was again administered another 24 h later, followed by a test of memory retention (extinction testing) and recovery (reinstatement testing). If prior reports of propranolol-induced post-reactivation amnesia are due to a retrieval deficit, we should observe amnesia, as indicated by a lack of differential FPS responding during retention and reinstatement testing, in group PrPl only. Re-administration of propranolol should then reverse the amnesia in group PrPr. However, if a storage deficit is the cause for the previously reported amnestic effect of propranolol, we should observe amnesia in groups PrPl and PrPr alike; amnesia should not be undone by the re-administration of propranolol (group PrPr). We did not expect any differences between groups in differential skin conductance response (SCR) or US expectancies, given that post-reactivation propranolol administration in humans has been shown to affect only emotional and not declarative aspects of memory (Soeter and Kindt, 2010; Sevenster et al., 2012; Cogan et al., 2018).

## Materials and Methods

### Pre-registration

The experimental procedures and statistical analysis plan were pre-registered on AsPredicted^1^.

### Participants

One hundred and eight volunteers were originally recruited to participate in the study through the university data pool, flyers and social media. They were first asked to complete the Anxiety Sensitivity Index (ASI) online to determine eligibility for participation. Those with a score of 26 or above were not allowed to participate further (*n* = 23), leaving 85 participants that entered further screening. One was subsequently excluded due to the presence of a current or previous medical condition that contra-indicated the use of propranolol (the complete list of medical exclusion criteria and contra-indications to propranolol use can be found in the Supplementary Material). Heart rate and blood pressure were repeatedly measured throughout the experiment to ensure that they did not fall below the cutoff values contra-indicated for propranolol use, leading to the exclusion of 2 more participants during testing. Those that did not complete all three days of the experiment were also excluded (*n* = 5), as were 4 more participants due to an error in the pharmacy logs that prevented us from obtaining their medication on time. Finally, those who did not exhibit successful fear learning by the end of the acquisition phase, as demonstrated by positive non-zero CS+/CS- FPS differentiation over the last block of acquisition, were also excluded (*n* = 13). The final sample included 60 participants (44 women), aged 18–40 (*M* = 21.93, *SD* = 4.06). All participants gave written informed consent in accordance with the Declaration of Helsinki and were reimbursed with 50 euros or partial research credits for their participation. The study was granted full ethical approval by the UZ Leuven Medical Ethics Committee.

### Stimuli

#### Conditioned Stimuli (CSs)

The conditioned stimuli were two images of spiders selected from the International Affective Picture System (IAPS # 1200 and 1201; Lang et al., 1997), which had been previously used in similar research (Kindt et al., 2009; Soeter and Kindt, 2010; Sevenster et al., 2012). Allocation of the images to the role of CS+ and CS- was counterbalanced across participants. The pictures had a resolution of 1024 × 768 pixels and measured approximately 22.5 × 16.5 cm on the screen.

#### Unconditioned Stimulus (US)

A mild 2-ms electrical shock to the wrist served as the US and was delivered to the top of the wrist of the dominant hand through a stimulating bar electrode, composed of two 8-mm stainless steel electrodes with an inter-electrode distance of 30 mm (Digitimer, Hertfordshire, United Kingdom). The shock was generated by a Digitimer DS7A constant-current stimulator (Hertfordshire, United Kingdom), controlled by Affect 4.0 software (see Procedure section). Using a shock work-up procedure, participants were given the opportunity to set their own individual shock intensity at a level that felt “uncomfortable, but not painful” (see Table 1 for average selected shock intensities per group). Once decided upon, this intensity was used throughout the experiment.

**Table 1:** Participant characteristics per group.

	Groups			
	PrPr *M* (SD)	PrPl *M* (SD)	PlPl *M* (SD)	NR *M* (SD)
Age (years)	22.27 (3.54)	22.27 (4.33)	21.53 (2.45)	21.67 (5.64)
Spider fear	43 (23.96)	45 (25.18)	37.20 (17.18)	41.07 (25.47)
Anxiety sensitivity	14.67 (6.40)	15.40 (6.15)	15.27 (6.39)	15.33 (4.94)
Trait anxiety	35.33 (7.93)	35.13 (8.43)	37.47 (6.66)	37.13 (13.21)
State anxiety	32.80 (6.97)	34.87 (6.90)	33.53 (5.90)	33.87 (7.81)
US intensity (mA)	31.13 (23.13)	33.53 (21.38)	28.20 (16.11)	30.47 (16.81)
US rating	8.23 (1.29)	8.23 (1.21)	8.71 (0.92)	9.07 (1.37)


### Subjective Assessments

#### Ratings and US Expectancies

Upon completion of the acquisition phase, participants were asked to retrospectively rate the unpleasantness induced by the US and the startle probes. Ratings were obtained using an 11-point scale ranging from “not unpleasant” (0) to “very unpleasant” (10). Additionally, they rated the intensity and surprisingness of the US and the startle probes, and the effort required to endure them, using a 5-point scale ranging from “light” (1) to “very strong” (5). Throughout the experiment, participants were asked to indicate their expectancy of the US using an 11-point scale ranging from “certainly no electric stimulus” (-5), over “uncertain” (0), to “certainly an electric stimulus” (5). This scale was presented at the bottom of the screen upon the onset of each CS presentation. Participants had 7 s to indicate their expectancy, allowing them enough time to respond before the startle probe was presented. In case participants did not respond within the 7-s window, the data for the trial were recorded as missing. Across all days, data were missing for 1.75% of the trials.

#### Questionnaires

The Anxiety Sensitivity Index (ASI; Peterson and Heilbronner, 1987) was used to assess participants’ tendency to respond fearfully to anxiety-related symptoms. The Fear of Spiders Questionnaire (FSQ; Szymanski and O’Donohue, 1995) was used to assess general level of spider phobia. Finally, state and trait anxiety were measured using the State and Trait Anxiety Inventory (STAI-S/STAI-T; Spielberger et al., 1977).

### Drug Treatment

#### Propranolol/Placebo

Propranolol HCl was administered orally in a 40-mg dose. Placebo pills identical in shape, size, and color were manufactured by a medical laboratory (Wolfs, Zwijndrecht, Belgium), and were also administered orally. As in previous studies, participants were asked to refrain from eating, drinking, smoking, and exercising prior to drug administration (Soeter and Kindt, 2010, 2011). Those instructions were previously given in relation to the collection of saliva samples and retained here for any influence they might have on the absorption of propranolol. Additionally, at the end of the second and third day of testing participants were asked to indicate whether they believed having received propranolol or placebo that day, through a simple question (“Which pill do you believe you received today, propranolol or placebo?”).

#### Heart Rate and Blood Pressure

Heart rate (HR) and blood pressure (BP) were measured with an electronic blood pressure monitor attached with a cuff to the left upper arm (Omron, M2 IntelliSense, Hoofdorp, Netherlands). These measurements were obtained once prior to the start of the study on Day 1, and at the beginning and end of Day 2 and 3.

### Psychophysiological Measures

#### Fear-Potentiated Startle

FPS was measured through electromyography (EMG) of the right orbicularis oculi muscle. Two 4-mm Ag/AgCl electrodes filled with conductive electrolyte gel (Microlyte, Coulbourn Instruments, Holliston, MA, United States) were placed 1 cm below the pupil and 1 cm below the lateral canthus. A third (ground) electrode was placed on the forehead (Blumenthal et al., 2005). Acoustic startle probes (40 ms white noise, 100 dBA) were presented binaurally through headphones (Sennheiser HD 202). The EMG signal was amplified using an isolated bioamplifier with band-pass filter (Lablinc v75-04, Coulbourn Instruments) with a high pass filter of 13 Hz and a low pass filter of 500 Hz. The signal was sampled at 1000 Hz and was rectified and smoothed online at a time constant of 20 ms, using a 4-channel integrator (Lablinc v76-24, Coulbourn Instruments). The analog output was digitized by a 16-bit AD converter (National Instruments, NI-6221, Austin, TX, United States). FPS data was further processed offline using the software package Psychophysiological Analysis (PSPHA) (de Clercq et al., 2006). Blink amplitude was determined by subtracting a 20-ms baseline (0–20 ms following probe onset) from the peak response in a 21–200 ms window following probe onset. We did not observe any non-responders with respect to startle potentiation, yet trials with excessive movement or occurrence of a spontaneous blink at the time of probe presentation were excluded, resulting in a 7.2% loss of data. To standardize the data, means and standard deviations from the first day were used to calculate within-participant *Z*-scores. Due to a technical malfunction on one of the testing days, the responses of four participants could not be standardized using the data of the first session. For those participants, the data of the second and third day were used to standardize their FPS. Their pattern of responding was compared to the pattern of the raw data, and did not deviate in any way.

#### Skin Conductance Response

SCR was recorded using a pair of disposable, pre-gelled 8-mm Ag/AgCl electrodes (Biopac Systems, Goleta, CA, United States) attached to the palm of the non-dominant hand. The signal was measured at 200 Hz with an isolated skin conductance coupler (LabLinc v71-23, Coulbourn Instruments). The raw analog signal was digitized by a 16-bit AD converter (National Instruments, NI-6221). Offline data extraction was completed with MATLAB. SCR amplitude was determined by subtracting the average of a 2-s baseline (prior to stimulus onset) from the maximum response in a 0–7 s window following stimulus onset. All responses were kept in the analysis, and SCR data were *Z*-transformed analogously to FPS responses. A technical problem with our SCR module during the last three months of testing affected the data of 9 participants, and rendered them unusable. Due to the uneven sample distribution this caused in our data, SCR analyses will be reported only in the supplement.

### Procedure

The study was composed of three consecutive testing sessions, each about 24 h apart (±2 h) [protocol adapted from Kindt et al. (2009)]. The first session lasted 1 h, while the second and third sessions lasted 2 h each. Every day began with a brief medical screening, where HR and BP were measured, followed by attachment of the FPS, SCR, and shock electrodes. Then, a habituation phase was conducted where 10 Noise Alone (NA) startle probes were presented with a 15–25 s inter-trial interval (ITI), in order to stabilize baseline startle reactivity. Throughout the experiment, order of trial presentations was randomized in blocks of three stimuli (CS+, CS-, and NA), so that no trial type was presented more than two times in a row. Affect 4.0, a dedicated software package for psychological experiments, was used for stimulus presentation and psychophysiological recordings (Spruyt et al., 2009).

#### Acquisition

On the first day, all participants completed an identical acquisition phase. At the start of the session, after obtaining informed consent, medical exclusion criteria were checked and vitals were measured. Then the STAI-S, STAI-T, and FSQ were administered, followed by electrode placement and the shock work-up procedure (see above). Participants were then instructed that they would see two images of spiders and that one of them would always be followed by a shock, while the other one would never be followed by a shock (Sevenster et al., 2013). Additionally, they were told that based on stimulus presentation, they should learn to predict whether a shock would occur, and that it was important to remember this information for the next two sessions. Finally, they were instructed how to indicate their US expectancy on the scale. The acquisition phase consisted of 6 CS+ (100% reinforced), 6 CS-, and 6 NA trials. CS trials had a duration of 8 s, while NA trials had a duration of 40 ms (same as the startle probe). Startle probes were presented 7 s after CS onset and were followed by the US 500 ms later. All trials had a variable 15–25 s ITI (*M* = 20 s). Upon completion of the acquisition phase, the STAI-S was administered and ratings of the US and startle probes were obtained. Finally, participants were asked to verbally state which stimulus was followed by shock in the acquisition phase and explicitly instructed once again to remember this information for the next two sessions.

#### Reactivation and Drug Administration

Prior to the second session, an external collaborator not involved in the testing randomized participants into four groups matched on age, gender, trait anxiety (STAI-T), ASI and FSQ. The session began with HR and BP measurements, followed by the administration of the STAI-S. After electrode attachment, the instructions of the first day were reminded and the habituation phase commenced. In three of the four groups, a single presentation of the CS+ without shock was used to reactivate the conditioned fear memory, followed by an NA trial. Following the reactivation phase, electrodes were detached and Groups PrPr and PrPl were administered 40 mg Propranolol HCl, while group PlPl received a placebo. The fourth group (Group NR) received 40 mg Propranolol HCl in the absence of prior memory reactivation. All participants waited in the lab for 90 min following drug administration, during which time they were allowed to read some magazines. At the end of the session HR and BP were measured again, the STAI-S was re-administered, and participants were asked whether they thought they had received propranolol or placebo.

#### Drug Re-administration, Memory Retention and Reinstatement Testing

At the start of the final session, HR and BP were measured and 40 mg Propranolol HCl was re-administered to the PrPr group whereas all other groups received placebo. Participants waited in the lab for 60 min, in order for the propranolol to approach its peak plasma level by the time of memory retention testing (Gilman and Goodman, 1996). After filling in the STAI-S, electrodes were attached and the instructions were reminded, but this time stating only that the same images would be presented as on the previous days, without any contingency information. Following the habituation phase, memory retention was examined in an extinction session involving the presentation of 12 CS+, 12 CS-, and 12 NA trials. This phase not only served to examine memory expression, but also to extinguish conditioned fear responding. Following the extinction session, participants remained in the lab, uninstructed, looking at a black screen with a white fixation cross for 10 min. Ten minutes after the last extinction trial, 3 unsignalled USs were administered, followed after 1 min by the presentation of another 4 CS+, 4 CS-, and 4 NA trials, all unreinforced, to test for sensitivity to reinstatement. At the end of the session, participants were asked to once again fill in the STAI-S and HR and BP were measured. The experiment concluded with participants again being asked whether they thought they had received propranolol or placebo that day.

### Statistical Analyses

Questionnaire data, US and startle probe ratings, and baseline HR and BP measurements were analyzed using one-way analysis of variance (ANOVA) with Group as between-subjects factor. Subsequent HR and BP measurements and STAI-S scores were analyzed using repeated-measures (rm) ANOVA with Group as between-subjects factor and Moment as within-subjects factor. Responses to the question regarding pill administration were subjected to a chi-squared test. After transformation, FPS and SCR outliers were determined for each day (*Z*-score > 3). Outliers and missing values were replaced by linear trend at point. Responses were then averaged over blocks of two trials (with the exception of the reactivation trial and the first trial of reinstatement testing) and subjected to rm-ANOVA with Group as between-subjects factor and Block (First, Last) and Cue [CS+, CS-, (NA)] as within-subjects factors. US expectancies were analyzed using rm-ANOVA with Group as between-subjects factor and Trial (First, Last) and Cue as within-subjects factors. When an interaction at the group level was found, follow-up rm-ANOVAs and independent samples *t*-tests were conducted to compare specific groups to each other. Greenhouse–Geisser corrections were applied in case of violation of sphericity. An alpha level of 0.05 was set for all analyses, which were executed using JASP version 0.8.6 (JASP Team, 2018).

## Results

### Participant Characteristics and Manipulation Checks

The four groups did not differ in age, *F*(3, 56) < 1, gender distribution, *X*^2^(3, *N* = 60) = 0.68, *p* = 0.88, education level, *X*^2^(6, *N* = 60) = 5.53, *p* = 0.48, or spider fear, *F*(3, 56) < 1. The individually selected US intensity was comparable between groups, *F*(3, 56) < 1, as was the subjective rating of US intensity, *F*(3, 56) = 1.68, *p* = 0.18, *$\eta_{p}^{2}$* = 0.08. Finally, we did not find any significant differences in baseline anxiety measures between the groups, either in ASI, trait anxiety, or state anxiety, all *F*(3, 56) < 1. For a complete overview of participant characteristics see Table 1.

The unpleasantness, intensity and surprise caused by the startle probes and the effort required to endure them did not differ between the groups, *F*(3, 56) < 1; *F*(3, 56) = 1.11, *p* = 0.35, *$\eta_{p}^{2}$* = 0.06; *F*(3, 56) < 1; *F*(3, 56) = 1.19, *p* = 0.32, $\eta_{p}^{2}$ = 0.06, respectively. The unpleasantness of the US and the effort to endure it did not differ between the groups either, *F*(3, 56) < 1; *F*(3, 56) = 1.27, *p* = 0.29, $\eta_{p}^{2}$ = 0.06, respectively. We did find marginal group differences in the intensity and surprisingness of the US, *F*(3, 56) = 3.03, *p* = 0.04, $\eta_{p}^{2}$ = 0.14; *F*(3, 56) = 2.71, *p* = 0.05, $\eta_{p}^{2}$ = 0.13, respectively. Follow-up *t*-tests revealed that the PrPr group rated the intensity of the shock (*M* = 2.93, *SD* = 0.59) slightly lower than the PrPl group (*M* = 3.33, *SD* = 0.49), *t*(28) = 2.02, *p* = 0.05, and the NR group (*M* = 3.47, *SD* = 0.52), *t*(28) = 2.63, *p* = 0.01, but not significantly different from the PlPl group (*M* = 3.20, *SD* = 0.41), *t*(28) = 1.43, *p* = 0.17. The exact same pattern was observed for the ratings of surprise, with the PrPr group scoring lower (*M* = 3.33, *SD* = 0.82) than the PrPl group (*M* = 3.93, *SD* = 0.70), *t*(28) = 2.16, *p* = 0.04, and the NR group (*M* = 4.07, *SD* = 0.70), *t*(28) = 2.64, *p* = 0.01, but not significantly different from the PlPl group (*M* = 3.80, *SD* = 0.78), *t*(28) = 1.61, *p* = 0.12. These ratings were obtained retrospectively following completion of the acquisition phase; all experimental procedures were identical for all participants up to that point. As the subjective ratings of the chosen US intensity following the shock work-up procedure were similar for all groups, these marginal differences in retrospective ratings should not have influenced the degree of learning during training.

No differences were found between groups in the evolution of state anxiety from prior to after acquisition, main effect of moment, *F*(1, 56) < 1, group × moment interaction, *F*(3, 56) = 1.30, *p* = 0.28, $\eta_{p}^{2}$ = 0.07, implying that all groups experienced the conditioning procedure similarly. To evaluate if drug (re-)administration had an effect on state anxiety, we compared STAI-S scores from the beginning of the second session (prior to retrieval; i.e., moment 3) to the end of the third session (i.e., moment 6) and found a significant group × moment interaction, *F*(3, 56) = 2.93, *p* = 0.04, $\eta_{p}^{2}$ = 0.14, suggesting differing subjective anxiolysis in the separate groups. Numerically, STAI-S scores in the PrPr group decreased from moment 3 (*M* = 32.93, *SD* = 7.91) to moment 6 (*M* = 29.93, *SD* = 8.20), while they increased in all other groups (PrPl_3_: *M* = 33.33, *SD* = 6.04, PrPl_6_: *M* = 36.13, *SD* = 11.19; PlPl_3_: *M* = 31.67, *SD* = 6.06, PlPl_6_: *M* = 33.40, *SD* = 6.45; NR_3_: *M* = 33.40, *SD* = 10.11, NR_6_: *M* = 36.80, *SD* = 10.84). This suggests that the PrPr group was less anxious by the end of the study; however, follow-up *t*-tests did not reach statistical significance.

Baseline HR and BP (systolic/diastolic) did not differ between the groups prior to the start of the study, main effect of group, *F*(3, 56) < 1, for all three comparisons. To examine the objective effects of drug administration, HR and BP were compared from the beginning to the end of the session, for Days 2 and 3 separately (see Table 2). HR decreased on Day 2 following pill administration, main effect of moment, *F*(1, 56) = 149.38, *p* < 0.001, *$\eta_{p}^{2}$* = 0.77; the degree of decline differed between groups, group × moment interaction, *F*(3, 56) = 8.74, *p* < 0.001, $\eta_{p}^{2}$ = 0.32. As expected, the PlPl group exhibited the smallest decline in HR, *t*(14) = 2.89, *p* = 0.01, while HR in all other groups decreased strongly, PrPr: *t*(14) = 7.15, *p* < 0.001; PrPl: *t*(14) = 8.20, *p* < 0.001; NR: *t*(14) = 5.90, *p* < 0.001. A similar main effect of moment was observed for systolic BP, *F*(1, 56) = 88.40, *p* < 0.001, $\eta_{p}^{2}$ = 0.61, and even though the interaction at the group level did not reach significance, *F*(3, 56) = 2.12, *p* = 0.1, $\eta_{p}^{2}$ = 0.10, the PlPl group again showed the smallest decline. The analysis of diastolic BP revealed comparable effects, main effect of moment, *F*(1, 56) = 17.76, *p* < 0.001, $\eta_{p}^{2}$ = 0.24; group × moment interaction, *F*(3, 56) = 3.62, *p* = 0.03, $\eta_{p}^{2}$ = 0.32, with all the propranolol groups showing a decline in diastolic BP whereas diastolic BP did not change from the beginning to the end of the session in group PlPl, *t*(14) = 0.20, *p* = 0.84.

**Table 2:** Heart rate and blood pressure before and after pill administration on Day 2 and Day 3.

			Day 2			Day 3		
			HR	Systolic BP	Diastolic BP	HR	Systolic BP	Diastolic BP
Groups	PrPr	Before	71.67 (8.22)	115.3 (7.76)	69.67 (6.53)	68.27 (9.38)	113.8 (8.51)	69.47 (5.62)
		After	57.40 (6.32)	103.9 (7.55)	67.00 (6.01)	57.40 (8.58)	100.9 (9.01)	66.13 (5.55)
		*t*	7.15^∗∗∗^	6.68^∗∗∗^	1.92^∗^	3.15^∗∗^	5.33^∗∗∗^	2.03^∗^
	PrPl	Before	82.27 (12.16)	116.3 (11.63)	73.80 (6.60)	73.07 (11.14)	115.2 (9.59)	72.67 (7.38)
		After	59.67 (8.02)	102.7 (7.75)	68.93 (6.56)	62.07 (9.42)	106.7 (10.39)	71.73 (6.40)
		*t*	8.20^∗∗∗^	5.52^∗∗∗^	3.09^∗∗^	3.21^∗∗^	3.94^∗∗∗^	0.63
	PlPl	Before	70.67 (12.06)	115.9 (12.69)	70.80 (6.49)	74.40 (13.65)	116.9 (13.37)	70.47 (7.25)
		After	65.27 (9.84)	109.8 (11.96)	71.13 (7.24)	66.20 (10.49)	109.6 (15.50)	68.47 (6.36)
		*t*	2.89^∗∗^	2.51^∗∗^	–0.20	6.33^∗∗∗^	2.83^∗∗^	1.06
	NR	Before	77.53 (11.57)	122.8 (10.42)	78.67 (5.83)	74.67 (14.86)	117.6 (10.82)	72.47 (6.77)
		After	59.80 (6.61)	109.9 (10.66)	72.33 (6.80)	65.93 (8.49)	112.7 (7.87)	72.53 (7.30)
		*t*	5.90^∗∗∗^	4.87^∗∗∗^	3.53^∗∗^	2.39^∗∗^	2.40^∗∗^	–0.04
ANOVAs	F_Moment_		149.38^∗∗∗^	88.40^∗∗∗^	17.76^∗∗∗^	38.84^∗∗∗^	53.05^∗∗∗^	3.20^∗^
	F_Group_ ×_Moment_		8.74^∗∗∗^	2.12^∗^	3.26^∗∗^	0.22	2.16^∗^	0.71


*Mean values (SD) for heart rate (BPM) and systolic and diastolic blood pressure (mmHg), before and after pill administration, at the beginning and end of Days 2 and 3, by group. Paired t-tests by group (before vs. after) and rm-ANOVAs (group × moment) are marked for significance (^∗^*p* ≤ 0.1, ^∗∗^*p* ≤ 0.05, and ^∗∗∗^*p* ≤ 0.001).*

On Day 3, HR decreased in all groups from before to after pill administration, *F*(1, 56) = 38.84, *p* < 0.001, $\eta_{p}^{2}$ = 0.41, with no group differences, group × moment interaction, *F*(3, 56) < 1. Likewise, systolic BP decreased overall, *F*(1, 56) = 53.05, *p* < 0.001, $\eta_{p}^{2}$ = 0.49. While the greatest decrease was observed in the PrPr group, *t*(14) = 5.33, *p* < 0.001, the interaction at the group level revealed a trend only, *F*(3, 56) = 2.16, *p* = 0.1, $\eta_{p}^{2}$ = 0.10. Examining the course of diastolic BP, a rm-ANOVA at the group level did not yield significant effects, main effect of moment, *F*(1, 56) = 3.20, *p* = 0.08, $\eta_{p}^{2}$ = 0.05; group × moment interaction, *F*(3, 56) < 1. Inspecting each group separately, we observed a marginally significant decrease in diastolic BP in the PrPr group only, *t*(14) = 2.03, *p* = 0.06, while none of the other (placebo) groups exhibited a change in their levels from before to after pill administration. Taken together, the HR and BP patterns indicate that propranolol exerted its intended physiological effects on both days of administration.

Finally, the groups did not differ with respect to the pill they believed to have received in the second, *X*^2^(3, *N* = 60) = 3.41, *p* = 0.33, or third session, *X*^2^(3, *N* = 60) = 3.53, *p* = 0.32. Like in Kindt et al. (2009), across all groups and on both days, the majority of participants believed to have been administered placebo (Day 2: 73%, Day 3: 72%).

### US Expectancies

**FIGURE 2:**
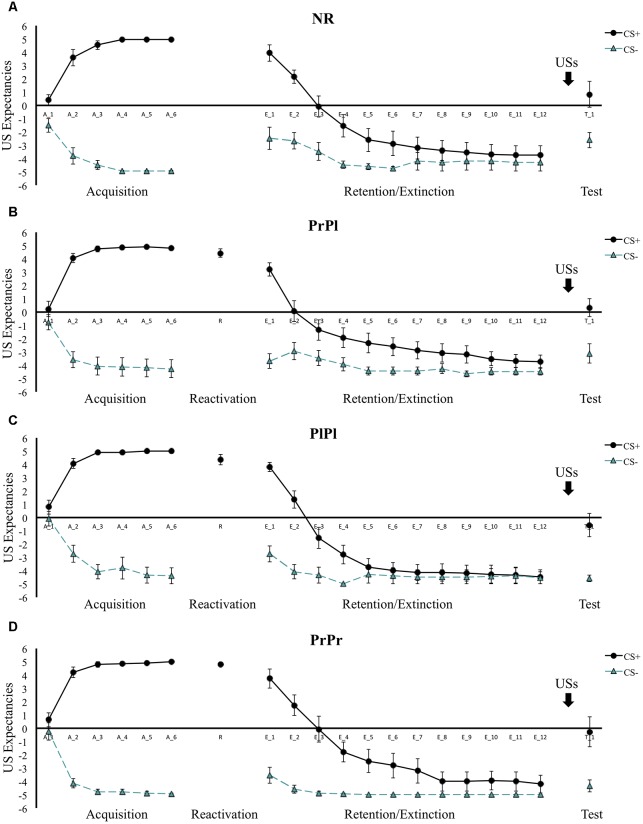
Mean US expectancies across all phases for **(A)** the NR group, **(B)** the PrPl group, **(C)** the PlPl group, and **(D)** the PrPr group. Error bars represent standard error of the mean.

Participants exhibited successful differential learning in the first session, indicated by a significant cue × trial interaction, *F*(1, 42) = 351.34, *p* < 0.001, $\eta_{p}^{2}$ = 0.89. On the last trial of acquisition, participants indicated a higher US expectancy for the CS+ than the CS-, *t*(58) = 37.49, *p* < 0.001. This pattern did not differ between the groups, group × cue × trial interaction, *F*(3, 42) < 1, suggesting similar acquisition of US expectancies across all participants (see Figure 2). During the second session, groups PrPr, PrPl, and PlPl responded comparably to the retrieval trial, main effect of group, *F*(2, 40) < 1. From the end of acquisition to the beginning of retention testing on Day 3, differential US expectancies decreased, cue × trial interaction, *F*(1, 53) = 25.36, *p* < 0.001, $\eta_{p}^{2}$ = 0.32, comparably across all groups, group × cue × trial interaction, *F*(3, 53) < 1, yet clear differential responding remained for all groups at the beginning of retention testing, main effect of cue, *F*(1, 54) = 179.70, *p* < 0.001, $\eta_{p}^{2}$ = 0.77; group × cue interaction, *F*(3, 54) < 1. Differential US expectancies were extinguished over the course of retention testing, cue × trial interaction, *F*(1, 54) = 153.93, *p* < 0.001, $\eta_{p}^{2}$ = 0.74, with no differences between the groups, group × cue × trial interaction, *F*(3, 54) < 1. From the last trial of extinction to the first trial of reinstatement testing, a significant cue by trial interaction emerged, pointing to differential reinstatement in US expectancies, *F*(1, 48) = 42.14, *p* < 0.001, $\eta_{p}^{2}$ = 0.47, that did not differ between the groups, group × cue × trial interaction, *F*(3, 54) < 1. In sum, in accordance with our hypothesis, propranolol administration did not affect declarative responding, as we observed retention of differential US expectancies at the beginning of the third session, as well as their reinstatement after successful extinction.

#### Fear-Potentiated Startle

**FIGURE 3:**
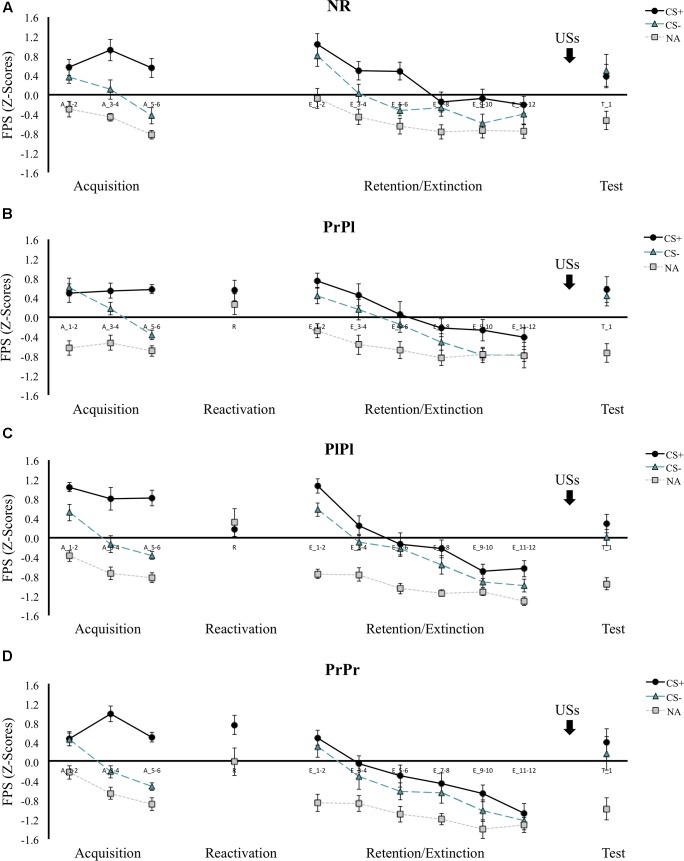
Mean FPS scores (*Z*-transformed) across all phases for **(A)** the NR group, **(B)** the PrPl group, **(C)** the PlPl group, and **(D)** the PrPr group. Error bars represent standard error of the mean.

Differential FPS responding increased from the first to the last block of acquisition, cue × block interaction, *F*(1, 56) = 48.19, *p* < 0.001, $\eta_{p}^{2}$ = 0.46, with participants displaying greater startle amplitudes to the CS+ than to the CS- in the last acquisition block, *t*(59) = 13.95, *p* < 0.001 (see Figure 3). This pattern did not differ between the groups, group × cue × block interaction, *F*(3, 56) < 1, nor did it change when the CS+ was compared to the NA rather than the CS-, cue × block interaction, *F*(1, 56) = 10.16, *p* = 0.002, $\eta_{p}^{2}$ = 0.15; group × cue × block interaction, *F*(3, 56) = 1.06, *p* = 0.37, $\eta_{p}^{2}$ = 0.05. On the second day, memory retrieval was seemingly not successful, main effect of cue, *F*(1, 42) = 2.80, *p* = 0.10, $\eta_{p}^{2}$ = 0.06. A closer look revealed that statistically, only the PrPr group responded more to CS+ than NA during the retrieval session, *t*(14) = 2.29, *p* = 0.04, while the PrPl and PlPl groups did not, *t*(14) = 1, *p* = 0.33; *t*(14) = 0.45, *p* = 0.66, respectively. Nevertheless, unlike the PlPl group that showed an opposite pattern (NA > CS+), the PrPl group exhibited numerically higher responses to the CS+ (*M* = 0.55, *SD* = 0.82) than to the NA (*M* = 0.27, *SD* = 0.84).

On the first block of retention testing, contrary to our hypotheses, differential FPS was intact in all the groups, main effect of cue, *F*(1, 56) = 13.09, *p* < 0.001, $\eta_{p}^{2}$ = 0.19; group × cue interaction, *F*(3, 56) < 1, although the degree of differentiation decreased from the end of acquisition to the beginning of retention testing, cue × block interaction, *F*(1, 56) = 56.31, *p* < 0.001, $\eta_{p}^{2}$ = 0.50, similarly in all groups, group × cue × block interaction, *F*(3, 56) < 1. Follow-up analyses revealed that whereas CS+ responding increased only modestly, main effect of block, *F*(1, 59) = 4.36, *p* = 0.04, $\eta_{p}^{2}$ = 0.07, CS- responding increased more considerably, main effect of block, *F*(1, 59) = 80.84, *p* < 0.001, $\eta_{p}^{2}$ = 0.58, suggesting increased fear generalization to the safe stimulus with the passage of time. When the same analysis was repeated with the CS+ versus the NA, a significant group × cue interaction emerged, *F*(3, 56) = 2.86, *p* = 0.045, $\eta_{p}^{2}$ = 0.13. NA responding remained stable from the end of acquisition to the beginning of retention in the PrPr and PlPl groups, *Z* = 49, *p* = 0.56; *t*(14) = 0.60, *p* = 0.56, respectively, but increased in the PrPl and NR groups, *t*(14) = 2.80, *p* = 0.01; *t*(14) = 3.54, *p* = 0.003, respectively. Increased baseline startle amplitudes have been suggested to reflect greater general state anxiety (Poli and Angrilli, 2015). Note that the PrPl and NR groups scored higher numerically on the STAI-S at the beginning of retention, although this effect did not reach statistical significance, main effect of group, *F*(3, 56) = 1.16, *p* = 0.33, $\eta_{p}^{2}$ = 0.06.

The augmented CS- responding at the beginning of retention testing obscured the observation of differential extinction learning from the beginning to the end of the retention phase, cue × block interaction, *F*(1, 56) < 1; group × cue × block interaction, *F*(3, 56) < 1. However, when the CS+ was compared to NA, a significant extinction pattern emerged from the first to the last block, cue × block interaction, *F*(1, 56) = 42.95, *p* < 0.001, $\eta_{p}^{2}$ = 0.43; group × cue × block interaction, *F*(3, 56) < 1, suggesting that extinction had taken place. Of note, in both analyses, we observed a main effect of group, *F*(3, 56) = 4.02, *p* = 0.01, $\eta_{p}^{2}$ = 0.18; *F*(3, 56) = 7.42, *p* < 0.001, $\eta_{p}^{2}$ = 0.29, respectively, due to the PrPr group exhibiting diminished responding to all cues at both time points. To further examine whether extinction learning occurred, and to verify the attenuation of FPS in the PrPr group unconfounded by fear generalization at the beginning of retention testing, we compared the last block of acquisition with the last block of extinction and observed a decline in differential responding, cue × block interaction, *F*(1, 56) = 45.36, *p* < 0.001, $\eta_{p}^{2}$ = 0.45, that did not differ between the groups, group × cue × block interaction, *F*(3, 56) < 1, suggesting successful extinction. Further, we found a significant group × block interaction, *F*(3, 56) = 2.99, *p* = 0.04, $\eta_{p}^{2}$ = 0.14, and a main effect of group, *F*(3, 56) = 3.48, *p* = 0.02, $\eta_{p}^{2}$ = 0.16, pointing to weaker FPS responding during extinction in the PrPr group. To confirm those observations, the PrPr group was compared to each of the other three groups separately during the course of extinction. A group effect emerged in each of those comparisons, main effect of group, PrPr versus PrPl: *F*(1, 28) = 4.59, *p* = 0.04, $\eta_{p}^{2}$ = 0.14; PrPr versus PlPl: *F*(1, 28) = 4.86, *p* = 0.04, $\eta_{p}^{2}$ = 0.15; PrPr versus NR: *F*(1, 28) = 8.59, *p* = 0.007, $\eta_{p}^{2}$ = 0.24.

In the last block of the extinction phase, there were no group differences in the degree of differential responding, group × cue interaction, *F*(3, 56) < 1, but the PrPr group demonstrated an attenuation in their startle responding, whether considering CS+/CS- responding, main effect of group, *F*(3, 56) = 4.00, *p* = 0.01, $\eta_{p}^{2}$ = 0.18, or CS+/NA responding, main effect of group, *F*(3, 56) = 6.38, *p* < 0.001, $\eta_{p}^{2}$ = 0.26. Comparing the last block of extinction to the first trial of reinstatement, we saw a non-differential (cue × time interaction, *F*(1, 56) < 1) increase in responding to all cues, main effect of cue, *F*(1, 56) = 5.97, *p* = 0.02, $\eta_{p}^{2}$ = 0.10; main effect of time, *F*(1, 56) = 79.04, *p* < 0.001, $\eta_{p}^{2}$ = 0.59, in all groups, group × cue × time, *F*(3, 56) < 1; main effect of group, *F*(3, 56) = 2.07, *p* = 0.12, $\eta_{p}^{2}$ = 0.10.

In contrast to our hypothesis and to previous reports, propranolol administration after reactivation did not affect differential FPS responding during memory retention testing, nor did it prevent reinstatement after extinction (PrPl group). We did observe an acute effect of propranolol administration on fear memory expression during extinction learning only, as the PrPr group exhibited attenuated startle responding throughout memory retention testing, yet similar sensitivity to reinstatement as the other groups.

## Discussion

In the present study, we investigated the effects of post-reactivation propranolol administration on fear memory expression. Specifically, we aimed to examine whether previous observations of propranolol-induced post-reactivation amnesia in humans reflect a disruption of reconsolidation, and therefore a loss of the original memory (storage deficit), or are due to incongruency of the internal state during memory retention testing with the drug-induced state created upon memory reactivation preventing successful retrieval (retrieval deficit). Participants received differential fear conditioning on the first day, and 24 h later, their memory was either reactivated (groups PrPr, PrPl, PlPl) or not (group NR) before being administered 40 mg Propranolol HCl (groups PrPr, PrPl, NR) or placebo (group PlPl). On the third day, propranolol (group PrPr) and placebo (all others) were re-administered 60 min prior to an extinction session (retention testing) that was followed by a test of fear recovery (i.e., reinstatement). We did not observe any effects of post-reactivation propranolol administration on memory retention or recovery, which prevented us from obtaining conclusive evidence in favor of either the storage or the retrieval deficit account of post-reactivation amnesia. We did observe an attenuation of FPS responding during retention testing in the PrPr group, suggesting that propranolol administration acutely affects fear memory expression under extinction. Below we discuss our findings in relation to storage and retrieval deficit accounts of post-reactivation amnesia and then consider acute effects of propranolol on extinction.

While our experimental protocol aimed to differentiate between the two rivaling accounts of retrograde amnesia, our findings are challenging to interpret from both views. According to the storage deficit account, propranolol administration following memory destabilization impairs the protein synthesis cascade that is assumed to be critical for a memory trace to become stable again (Kindt et al., 2009; Soeter and Kindt, 2010; Kindt and Soeter, 2018). The reconsolidation process is thus disrupted and memory re-storage is prevented (Dȩbiec and LeDoux, 2004). If this were the case, in our study we would have expected the PrPl and PrPr groups to exhibit reduced CS+/CS- differentiation at the beginning of retention and reinstatement testing, relative to the PlPl and NR groups. On the other hand, from a retrieval-deficit perspective, according to the integration hypothesis, any pharmacological intervention applied at the time of memory reactivation should be incorporated with the initial memory trace, producing state-dependency of the memory (Gisquet-Verrier et al., 2015). From this view, we would expect to observe amnesia in the PrPl group but not in the PrPr group, as here the amnesia would be lifted upon re-administration of propranolol. However, as we did not observe the intended amnestic effects following post-reactivation propranolol administration (i.e., group PrPl) to begin with, we cannot know whether re-administration of propranolol would have reversed an amnesia that was not attained in the first place.

It is possible that we failed to observe an amnestic effect of propranolol simply because our protocol was not successful in triggering the reconsolidation process. Ample research has demonstrated the importance of a prediction error (PE) at the time of memory retrieval in order for a memory trace to destabilize and reconsolidation to be induced (Pedreira et al., 2004; Forcato et al., 2009; Sevenster et al., 2012, 2013, 2014). An optimal level of PE, defined as the mismatch between what a participant expects to happen during the reactivation session (based on the retrieved memory) and what actually occurs (the events that happen during the retrieval experience), is suggested to be a critical and decisive factor for the induction of memory destabilization. Too little PE is proposed to result in mere memory retrieval without reactivation, while excessive PE promotes the formation of a novel memory trace instead of reactivation of an existing trace (Suzuki et al., 2004; Osan et al., 2011; Sevenster et al., 2014). It is in principle possible that we did not elicit a sufficient amount of PE during reactivation, preventing the induction of memory malleability. Note, however, that our acquisition and reactivation procedure (and thus, arguably, the degree of PE during retrieval) was identical to the one used in previous reports of successful post-reactivation amnesia following propranolol administration (Kindt et al., 2009; Soeter and Kindt, 2010, 2011; Sevenster et al., 2012; but see Bos et al., 2014; Schroyens et al., 2017). From our Day 2 FPS data, it may seem that we failed to elicit strong conditioned fear responding to the CS+ in some groups, implying that the memory trace might not have been successfully retrieved. Yet, when examining each group separately, this seems to apply to the PlPl group only, as the PrPr and PrPl groups exhibited numerically stronger responding to the CS+ than to the NA during reactivation and their CS+ responding remained stable from the end of acquisition over reactivation to the beginning of memory retention testing. It seems therefore safe to assume that in the groups of interest (PrPr and PrPl), CS+ presentation indeed effectively retrieved the consolidated fear memory trace, thus allowing for memory reactivation and the induction of reconsolidation. Note further that the dose of propranolol we used was identical to that of previous studies, as was the route of administration. Neither of those factors can therefore account for the divergent findings with previous studies.

Our protocol did deviate from most prior reports of propranolol-induced amnesia in humans in one potentially important detail – the reinforcement rate employed during conditioning. It has been suggested that strong memories are more difficult to disrupt using reconsolidation interference techniques (Dudai, 2004; Suzuki et al., 2004; Wang et al., 2009; Kindt, 2018). Further, a single unreinforced CS+ presentation during reactivation, suitable to produce memory destabilization after partial reinforcement, might yield too strong a PE (i.e., an excessive mismatch between conditioning and reactivation) after full reinforcement as used here, thereby promoting the formation of a new extinction memory rather than the destabilization of the acquisition memory (Reichelt and Lee, 2013; Sevenster et al., 2014). However, note that propranolol-induced post-reactivation amnesia has been observed using a full reinforcement schedule during acquisition before (Sevenster et al., 2013). Moreover, we see no evidence of excessive PE during memory retrieval in our data, as the decline in differential US expectancy ratings from the end of acquisition to the beginning of memory retention testing is very similar to that observed in previous reports where amnesia was obtained, and clearly smaller than the considerable decline observed under retrieval conditions that promote the formation of extinction memory (Sevenster et al., 2014).

A final factor that could influence our findings is participants’ trait anxiety. While high trait anxiety is not considered a boundary condition for the induction of reconsolidation *per se*, it has been suggested to be associated with lesser fear reduction after reconsolidation interference (Soeter and Kindt, 2013). In our sample, average STAI-T scores matched those of high trait anxiety samples in previous reports (Soeter and Kindt, 2013). Also, the combination of a relatively high-trait anxious sample and 100% reinforcement rate during acquisition might be responsible for the strong fear generalization to the CS- that we observed in all groups at the beginning of retention testing. At the start of the first two sessions of the study, participants were instructed that one stimulus would always be followed by the aversive outcome, while in reality, this happened during the first session only. For the second session, those instructions were given with the intent of triggering a PE that would induce destabilization of the conditioned memory trace. However, the fact that participants did not actually receive the US during the second session despite having been instructed that they would, might have been a cause of uncertainty and ambiguity on the part of the participants. Given the evidence of deficient safety learning in high trait anxious individuals (Gazendam et al., 2013) and indications of impaired discrimination learning in ambiguous situations in stress-sensitive individuals (Arnaudova et al., 2013), it is conceivable that the perceptual similarity between the two CSs promoted particularly strong generalization of fear to the CS- at the beginning of retention (extinction training) in the present sample (Lissek et al., 2008; Haddad et al., 2012).

While our findings failed to shed light on the mechanism governing propranolol-induced post-reactivation amnesia in humans, we did observe an acute effect of propranolol on extinction performance. Participants in group PrPr (the only group that received propranolol prior to retention testing) exhibited attenuated startle responses throughout extinction training yet similar sensitivity to reinstatement as the other groups, suggesting that propranolol exerted an overall, non-associative effect on fear memory expression. The influence of noradrenergic blockade on extinction learning in humans is largely unknown, with two published studies reporting opposing findings (Bos et al., 2012; Kroes et al., 2016). In the study of Kroes et al. (2016), participants that received 40 mg of propranolol 60 min prior to an extinction session exhibited a lack of differential SCR responding during extinction, as well as a lack of return of differential SCR responding when tested in the absence of the drug one day later. Of note, the overall level of SCR responses was not attenuated during extinction, unlike FPS responses in the present study. On the other hand, Bos et al. (2012) reported impaired extinction of US expectancies and impaired extinction retention as a result of propranolol administration prior to non-differential extinction training, whereas FPS and SCR were unaffected. In the animal literature, the few studies that have been conducted have yielded similarly mixed findings (Cain et al., 2004; Mueller et al., 2008; Rodriguez-Romaguera et al., 2009). In line with the present results, Rodriguez-Romaguera et al. (2009) reported that pre-extinction propranolol administration attenuated conditioned responding during extinction training in rats but preserved the return of fear obtained in a subsequent reinstatement procedure. Cain et al. (2004) found the opposite effect, with propranolol yielding an enhancement of fear when administered prior to spaced extinction trials (20 min ITI). Mueller et al. (2008) found yet another result, observing no effect of pre-extinction propranolol on within-session extinction performance but impaired extinction recall the following day.

The discrepancies in published findings regarding the effect of beta-blockade on extinction learning, extinction recall, and return of fear can be attributed in whole or in part to the different species and distinct paradigms, dosages and routes of administration used in those studies. It may therefore be premature to attempt to reach a definitive conclusion concerning the possible therapeutic effects of propranolol on emotional memory. At first glance, the findings of Kroes et al. (2016) carry translational promise and suggest that pre-extinction propranolol administration may reduce fear and prevent its subsequent recovery. However, their study followed an ABB design, meaning that fear conditioning was conducted in a different context from fear extinction and retention/reinstatement testing. If participants were to be re-introduced to the acquisition context, there may well be a renewal of fear responding (Bouton and King, 1983; Bouton, 2004), which would severely limit clinical applicability (i.e., patients may be expected to exhibit return of fear when confronted with the situation that induced their original fear or with an entirely novel situation). The observations of Kroes et al. (2016) are moreover in stark contrast not only with the present findings but also with those of Bos et al. (2012), where in both studies participants exhibited a recovery of fear when presented with reinstating USs. Finally, some evidence suggests that any positive acute effects of propranolol on fear responding during extinction may be offset by detrimental effects in the long run. Conditioned responding during extinction was attenuated in Rodriguez-Romaguera et al. (2009) and in the present study, suggesting an anxiolytic effect of propranolol that would in essence be helpful in a therapeutic setting, as it would reduce anxiety during exposure treatments. Yet, this beneficial effect may be only fleeting and reversed eventually, as propranolol administration impaired consolidation of extinction learning and facilitated persistence of fear in the studies of Bos et al. (2012) and Mueller et al. (2008). With only a handful of published studies investigating effects of noradrenergic blockade on extinction processes, more research will be vital to chart its parameter-dependent effects on processes that may variably enhance or attenuate fear in different species and various memory systems. In this regard, timing may be critical in modulating benefits of pre-extinction propranolol administration on fear memory expression and retention, given that Cain et al. (2004) observed an enhancement of fear as a result of propranolol administration when CS presentations were spaced during extinction training in mice, whereas the results of the present study in humans and the findings of Rodriguez-Romaguera et al. (2009) in rats point to an attenuation rather than an enhancement of conditioned responding during non-spaced extinction learning. Recovery of fear appeared to be unaffected in most studies (Mueller et al., 2008; Rodriguez-Romaguera et al., 2009; Bos et al., 2012), with the study of Kroes et al. (2016) as the only exception.

In sum, we failed to replicate previous observations of drug-induced amnesia following post-reactivation propranolol administration in humans. We did not detect any permanent effects of post-reactivation propranolol on fear memory retention nor its recovery after extinction. We did, however, obtain evidence for an acute effect of 40 mg Propranolol HCl on FPS responding during extinction learning, an effect that had not been documented before and critically extends prior findings about the influence of propranolol on extinction in humans and animals. Our results thus highlight the need for further investigation of the effects of beta blockade on (consolidation of) extinction learning and retention and of how those effects can be translated and integrated into an operational adjunct to exposure therapy to help those suffering from emotional disorders.

## Data Availability

De-identified processed data (excluding demographic and questionnaire data) are publicly available on the Open Science Framework at https://osf.io/4ypsq/.

## Author Contributions

AC, LVO, and TB designed the experiment and contributed to the interpretation of the results. AC and JW carried out the experiment and analyzed the experimental results. AC and TB wrote the manuscript. All authors reviewed and approved the final manuscript.

## Conflict of Interest Statement

The authors declare that the research was conducted in the absence of any commercial or financial relationships that could be construed as a potential conflict of interest.

## Footnotes

1 https://aspredicted.org/dd4ub.pdf
