# Supplementary material for: Acute but Not Permanent Effects of Propranolol on Fear Memory Expression in Humans
Source: Front Hum Neurosci. 2019 Feb 21;13:51. doi: 10.3389/fnhum.2019.00051 (PMC6394213; doi:10.3389/fnhum.2019.00051)
Supplement: Supplementary file 1 [file Data_Sheet_1.PDF]

Supplementary Material

Acute but not Permanent Effects of Propranolol on Fear Memory Expression in Humans

Anastasia Chalkia, Jeroen Weermeijer, Lukas Van Oudenhove, Tom Beckers*

***Correspondence:** Tom Beckers: tom.beckers@kuleuven.be

Medical exclusion criteria and contra-indications to propranolol use

Prior to the start of the first session, participants were screened for the presence of any of the following current or previous medical conditions that would exclude them from participating: (current diagnosis or history of) heart disease or other cardiovascular problems (e.g., palpitations, chest pain), heart disease in the immediate family (e.g., heart rhythm disorders, heart attacks <65 years, sudden death <60 years), asthma, lung disease or lung problems, low blood pressure, blackouts or fainting, diabetes, liver or kidney disorders, metabolic acidosis, excessive production of thyroid hormone, circulatory disorders, neurological disorders (e.g., epilepsy, convulsions), clinical depression, mania, psychosis, anxiety disorder, or other psychiatric disorder; presence of electronic implant (e.g., pacemaker); pregnancy (current or attempting); other serious medical conditions; pain or problems at the hands or wrists; request from the doctor to stay away from stressful situations; inability to perform moderate-effort exercise. Participants were also excluded for current medication use such as: medications that affect the heart, blood pressure, or blood sugar; medications against dizziness, migraines, tuberculosis, or psoriasis; gastric acid binders; anti-inflammatory analgesics; antidepressants, antipsychotics, or anxiety inhibitors; asthma medication; and illegal drugs (either current, past, or intended use). Finally, those with an allergy to Propranolol/Inderal, or with blood pressure below 90/60 mmHg or heart rate below 50 BPM at rest were also not allowed to participate.

SCR analyses

Due to technical problems with the recording equipment during the last months of the study, some SCR data were rendered unusable and therefore removed from further analysis. Nine participants were removed from all SCR analyses, and one participant (from the PrPl group) was removed from day 1 analyses only. The final SCR dataset includes 11 participants in the PrPr group, 13 in the PrPl and PlPl groups, and 14 in the NR group. During acquisition, differential responding did not increase from the first to the last block, cue x block interaction, $F(1, 46) = 2.40$, $p = .13$, $\eta_p^2 = 0.05$. Yet, when examining only the last block of acquisition, participants exhibited higher SCRs to the CS+ than to the CS-, main effect of cue, $F(1, 46) = 29.78$, $p < .001$, $\eta_p^2 = 0.39$, and this pattern did not differ between the groups, group x cue interaction, $F(3, 46) < 1$. Even though differential SCR responding did not increase during acquisition, higher responses to the CS+ by the end of the session point to successful learning across all groups. On the second session, all groups responded comparably to the CS+, main effect of group, $F(2, 34) = 1.15$, $p = .34$, $\eta_p^2 = 0.06$, suggesting that reactivation was similar for all groups.

On the first block of retention testing, similar to our FPS findings, differential SCR responding was intact across groups, main effect of cue, $F(1, 47) = 13.77$, $p < .001$, $\eta_p^2 = 0.23$; group x cue interaction, $F(3, 47) = 2.11$, $p = .11$, $\eta_p^2 = 0.12$. The degree of differentiation decreased from the end of acquisition to the beginning of retention, cue x block interaction, $F(1, 46) = 8.20$, $p = .006$, $\eta_p^2 = 0.15$, similarly in all the groups, group x

cue x block interaction, $F(3, 46) < 1$. Follow-up analyses examining each cue separately yielded a comparable pattern in SCR as was observed in FPS. While CS+ responding remained stable from the end of acquisition to the beginning of retention, main effect of block, $F(1, 46) < 1$, CS- responding increased, main effect of block, $F(1, 46) = 11.61$, $p = .001$, $\eta_p^2 = 0.20$. This increase was evident in all the groups, group x block interaction, $F(3,46) < 1$, again suggesting fear generalization to the CS- across all groups.

Comparing the beginning to the end of retention testing (extinction), we did not observe a decline in differential SCR responding, cue x block interaction, $F(1, 47) = 2.62$, $p = .11$, $\eta_p^2 = 0.05$, most likely due to the enhanced CS- responding at the beginning of extinction. To assess whether extinction learning took place, we therefore compared the last block of acquisition to the last block of extinction and observed a significant decline in differential SCR, cue x block interaction, $F(1, 46) = 17.71$, $p < .001$, $\eta_p^2 = 0.28$. This pattern differed slightly between the groups, group x cue x block interaction, $F(3, 46) = 2.86$, $p = .046$, $\eta_p^2 = 0.16$, suggesting distinct extinction learning patterns in SCR for the four groups. Follow-up analyses on each group separately revealed that the PrPl group did not actually fully extinguish their conditioned responding, cue x block interaction, $F(1, 11) < 1$, while all other groups did (PrPr: $F(1, 10) = 21.96$, $p < .001$, $\eta_p^2 = 0.69$; PlPl: $F(1, 12) = 16.98$, $p = .001$, $\eta_p^2 = 0.59$; NR: $F(1, 13) = 6.62$, $p = .02$, $\eta_p^2 = 0.34$).

We observed an increase in responding from the end of extinction to the beginning of reinstatement testing for both cues, main effect of time, $F(1, 47) = 39.25$, $p < .001$, $\eta_p^2 = 0.46$, with somewhat greater responding to the CS+, main effect of cue, $F(1, 47) = 4.90$, $p = .03$, $\eta_p^2 = 0.09$. Yet, our reinstatement procedure did not induce a significant differential fear recovery in any of the groups, cue x time interaction, $F(1, 47) = 1.76$, $p = .19$, $\eta_p^2 = 0.04$; group x cue x time interaction, $F(3, 47) = 2.27$, $p = .09$, $\eta_p^2 = 0.13$. This was also evident when we included the first trial of reinstatement only, as participants exhibited generalized fear responding across all groups, main effect of cue, $F(1, 47) = 3.87$, $p = .055$, $\eta_p^2 = 0.08$; group x cue interaction, $F(3, 47) < 1$. Even though we had to exclude an unequal number of participants from each group in our SCR analyses, our results are in line with our hypothesis, as we did not expect any effects of post-reactivation propranolol administration on SCR responding during extinction and reinstatement testing.
